# Electrofuels for Road, Rail, Maritime, and Aviation Sectors: Assessing the Potential Challenges and Opportunities for Decarbonization

**DOI:** 10.1021/acsomega.5c05945

**Published:** 2026-04-17

**Authors:** Megalingam Arumugampillai, Hariram Nediyirippil Prakasan, Shanmuga Priya Selvanathan, Sudhakar Kumarasamy

**Affiliations:** † Faculty of Mechanical and Automotive Engineering Technology, 65240 Universiti Malaysia Pahang Al-Sultan Abdullah, Pekan, Pahang 26600, Malaysia; ‡ Faculty of Civil Engineering Technology, Universiti Malaysia Pahang Al Sultan Abdullah, Paya Besar, Pahang 25150, Malaysia; § Integrated Centre for Green Development and Sustainability (ICFGS), ICFGS Foundation, Kazhimbram, Kerala 680568, India; ∥ Manipal Institute of Technology, 125853 Manipal Academy of Higher Education, Manipal, Karnataka 576104, India; ⊥ Automotive Engineering Centre, Universiti Malaysia Pahang Al Sultan Abdullah, Pekan, Pahang 26600, Malaysia; # Centre for Research in Advanced Fluid & Processes, Universiti Malaysia Pahang Al Sultan Abdullah, Gambang, Pahang 26300, Malaysia

## Abstract

Electrofuels (e-fuels) offer a decarbonization pathway for the hard-to-abate transport sectors of aviation, maritime, rail, and heavy-duty road transport by exploiting existing fuel infrastructure while eliminating fossil carbon emissions. Despite this advantage, commercial deployment remains constrained by prohibitive production costs (currently €3–6/L for e-diesel, €2–5/L for e-methanol), intensive energy requirements (∼50 kWh renewable electricity per liter), and systemic upscaling barriers including feedstock availability and carbon source purity. We critically examine sector-specific deployment potential, identifying aviation and maritime as priority markets where electrification alternatives remain limited while highlighting hybrid architectures (e-fuel/electric synergies) for rail and road applications. This review synthesizes recent technological advances demonstrating pathway efficiency improvements up to 70% through process intensification and advanced catalytic systems, notably CO_2_ hydrogenation selectivity exceeding 80% for e-kerosene synthesis. Life cycle assessments indicate emission reductions of 75–90% relative to fossil-fuel counterparts, contingent on fully renewable energy inputs. Economic modeling projects cost parity trajectories toward €1.5–3.0/L by 2030, driven by renewable energy scale-up and learning-curve effects in electrolyzer and synthesis technologies. Policy analysis highlights the necessity of carbon pricing mechanisms, renewable fuel mandates, and targeted R&D funding to derisk investment and accelerate market formation. Finally, we explain critical research gaps in large-scale system integration, sustainable carbon sourcing, and life cycle sustainability assessment methodologies. By addressing these multidimensional challenges, e-fuels can transition from niche demonstration to commercially viable bridge technologies on the path to fully sustainable transport ecosystems.

## Graphical abstract

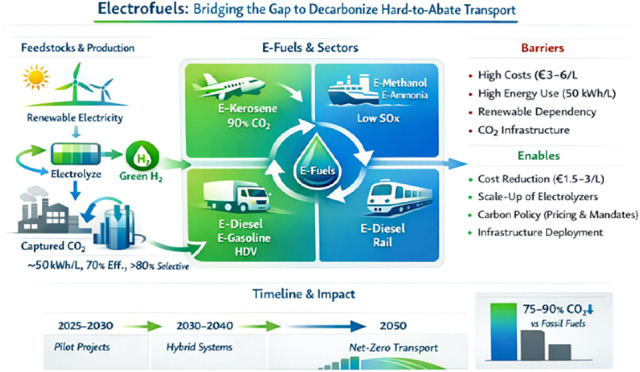

## 1 Introduction

The global transport sector is a major contributor to greenhouse gas emissions, necessitating urgent decarbonization strategies to meet sustainability goals.^1^ Among new solutions, e-fuels (electrofuels) have emerged as a tempting proposition with the ability to provide a carbon-neutral alternative to fossil fuels.^2^ Made by combining hydrogen through renewable energy-driven electrolysis with sequestered carbon dioxide (CO_2_), e-fuels can be retrofitted into all current internal combustion engine (ICE) vehicles and fuel distribution networks.^3^ This uncommon benefit makes e-fuels an important bridging solution to realize decarbonization without requiring significant changes to transportation systems. Electro-diesel is a renewable alternative to rail networks in areas where electrification is not possible.^4^ E-fuels are important across different modes of transport, including road, rail, shipping, and air. For road and rail transport, e-diesel and e-gasoline are utilized by heavy-duty trucks and long-distance freight vehicles, which gain high energy density and compatibility with existing fuel infrastructure.^5^ Likewise, e-diesel offers a renewable substitute for rail networks in areas where electrification is not viable due to economic or technological constraints.^6^ Marine and inland waterway transportation also suffer from severe decarbonization challenges, particularly for long-distance shipping.^7^ E-methanol and e-ammonia are emerging options now, with reduced storage costs, easier handling, and aligning with the International Maritime Organisation’s (IMO) emission targets.^8^ For aviation, where the energy demand and rigorous safety standards hinder electrification, e-kerosene is an emerging solution, allowing airlines to reduce emissions while tapping into established jet fuel infrastructure.^9^

In addition to emission reduction, e-fuels enhance supply chain resilience, energy security, and fuel flexibility in the decarbonization process.^10^ For net-zero-emission goals, e-fuels specifically provide a more circular carbon economy, where CO_2_ is captured and cycled back into energy. Yet, for large-scale applications, issues related to production costs, scalability, and access to renewable energy need to be addressed.^11–13^ Bridging the gap between current infrastructure and future clean energy technologies, e-fuels can be an integral part of a sustainable transportation network with policymakers’ support and technology advancements.^14,15^

### 1.1 Motivation of This Study

The transport sector is still a principal source of worldwide carbon emissions as it is based heavily on fossil fuels, especially in aviation, shipping, and heavy transport, where electrification is still unfeasible.^10,16,17^ Although battery-electric vehicles can be used for light-duty applications, the significant energy requirements and long ranges of hard-to-abate industries require other measures, such as e-fuels, to achieve deep decarbonization.^18,19^ Nonetheless, large-scale commercial deployment of e-fuel is challenging due to high energy use, limited availability of renewable electricity, and cost limitations for electrolysis and carbon capture technologies.^20^ Further discouraging the use of e-fuel in existing transportation is the lack of mature infrastructure and policy support that ensures investment in production facilities, supply chains, and regulatory infrastructure.^21,22^ Furthermore, the actual environmental benefits of e-fuels are unclear due to variations in manufacturing processes, fuels, and life cycle GHG emissions, prompting calls for uniform measurement methodologies and apples-to-apples comparison studies.^23^ Bridging these economic, technical, and policy-making hurdles is fundamental to realizing the full potential of e-fuels as a means of decarbonizing the transport sector.^24^ This review explores the pivotal role of e-fuels in shaping a sustainable transportation system, highlighting their potential to bridge the decarbonization gap, while addressing key challenges that hinder their full-scale adoption. By examining the position of e-fuels within the global energy transition, this study aims to contribute to the development of actionable strategies that pave the way for a cleaner and more sustainable future.

Overall, the main contributions of this study are described here:

- Identifies key economic, technical, and policy barriers limiting the large-scale adoption of e-fuels in hard-to-decarbonize transport sectors.
- Highlights gaps in previous review work by addressing the lack of consistent environmental impact assessments and proposing the need for standardized assessment methods.
- Provides strategic insights into how e-fuels can meet the global energy transition, particularly where electrification is not yet feasible.

### 1.2 Organization of This Study

This study is designed to thoroughly examine the role of e-fuels in decarbonizing sustainable transportation systems. The Introduction Section provides an overview of the need for alternative fuels and the prospects of e-fuels for reducing carbon emissions. The review of sustainable e-fuels covers their production processes, energy sources, and environmental impacts. The research further investigates sector-specific uses, including e-fuels for road and railways, e-fuels for marine and waterways, and e-fuels for aviation, based on their feasibility, efficiency, and technology levels. Techno feasibility and market barriers elaborate on mass adoption issues, including economic viability, infrastructural limitations, and policy barriers. The Future Scope and Recommendations Section outlines research directions, technological advancements, and policy initiatives to promote e-fuel adoption, and the Conclusion Section integrates major findings and their significance for sustainable mobility.

## 2 Sustainable E-Fuels: Low-Carbon Solutions for Hard-to-Decarbonize Sectors

E-fuels are not a silver bullet for the energy transition but present a vital stepping stone in decarbonizing hard-to-electrify sectors such as long-haul aviation, maritime shipping, and heavy industry.^25,26^ E-fuels can contribute toward reduced CO_2_ emissions if these are produced via renewable electricity and the use of captured CO_2_, but they are not necessarily carbon neutral, as there will be some embedded emissions in the whole life cycle of the fuel.^10^ The process of absorbing CO_2_ in fuel manufacturing and releasing it in combustion is not a closed carbon loop, particularly in areas such as aviation and transportation, where the released CO_2_ is not absorbed. E-fuels, consequently, should serve as a transition fuel and not a replacement for fossil fuels. They should retain their compatibility as a drop-in fuel.^27^ Governments and industries across the globe are stepping up investment in e-fuels, with several pilot projects and commercial-scale facilities already running or under construction, especially in Europe, where the EU’s Green Deal and Fit for 55 climate packages have made e-fuels a top decarbonization priority.^28,29^ E-fuels are a generic term for synthetic fuels that include e-diesel, e-gasoline, e-kerosene, e-methanol, e-ammonia, and e-hydrogen, which can power internal combustion engines and jet turbines directly with life cycle emissions substantially lower than traditional fuels.^4^ Advanced e-fuels like e-ammonia and e-hydrogen provide low-carbon shipping and heavy transport options, but challenges to infrastructure, storage, and energy efficiency will have to be addressed for widespread uptake.^23,30^ In aviation, e-kerosene is increasingly emerging as a green alternative to traditional jet fuel, enabling cleaner operations in areas where electrification is still not practical.^31^ With evolving technology, gains in electrolysis efficiency, direct air capture (DAC), and integration of large-scale renewable energy will be pivotal in lowering the cost of production and increasing the contribution of e-fuels in future energy security and climate mitigation strategies.^32,33^ The performance parameters of the e-fuels are compared with those of the fossil fuels used for the respective applications (Table 1). More specifically, the e-diesel, e-gasoline, and e-kerosene alternatives are compared against their fossil-fuel counterparts; the e-methanol and e-ammonia alternatives are compared against their fossil-fuel counterparts or conventional shipping fuels, whereas the e-hydrogen alternative is compared against the fossil-fuel (gray) hydrogen alternative.^34^

**Table 1:** Comparison of E-Fuels vs Conventional Fossil Fuels

parameter	e-diesel	e-gasoline	e-kerosene	e-methanol	e-ammonia	e-hydrogen
formula^23,30^	C_17_H_36_	C_8_H_18_	C_12_H_26_	CH_3_OH	NH_3_	H_2_
energy density^3,4^	35–39 MJ/L	32–35 MJ/L	34–37 MJ/L	15–16 MJ/L	18–19 MJ/L	120 MJ/kg (33.3 kWh/kg)
production^4,5^	RWGS + FT synthesis using renewable H_2_ + captured CO_2_	RWGS + FT synthesis using renewable H_2_ + captured CO_2_	RWGS + FT synthesis using renewable H_2_ + captured CO_2_	renewable H_2_ + captured CO_2_	Haber–Bosch process using green H_2_	water electrolysis using renewable electricity
Storage^6^	liquid (easy)	liquid (under pressure)	liquid (stable)	liquid (−97 °C)	liquid (−33 °C)	cryogenic (−253 °C)
CO_2_ reduction (%)^7^	up to 78	up to 65	up to 80	up to 95	≈100 (no direct CO_2_ emissions at use)	≈100 (no direct CO_2_ emissions at use)
emissions (gCO_2_/MJ)^35,36^	5–40	6–45	8–50	2–25	5–35	5–40

Fasihi et al. (2019)^37^ and Singh et al. (2022)^20^ study suggests the potential for an overall reduction of CO_2_ emissions when produced with low-carbon electrons and CO_2_ capture, even if currently not being carbon neutral because of the greenhouse gases embedded in the use of energy and the CO_2_ extraction. Though the process involves the release of CO_2_ in the burning of fuel, it also involves the trapping of CO_2_ in fuel generation, but this does not indicate a cycle, especially where the aviation or transport industry cannot reuse the CO_2_. Despite this, e-fuels do provide promise for difficult-to-abate sectors where direct electrification is limited by their energy density and range issues.^38^ However, the challenge of scalability and economic viability has been a problem, as discussed by Gabrielli et al. (2020), who state that the high-power requirements of electrolysis and carbon capture, along with the currently high cost of renewable electricity, make e-fuels currently more expensive than regular fossil fuels.^39^ Despite this, it is expected that further advancements in technology and economies of scale will enhance cost competitiveness.^38,39^ This is not to say that technological innovation in renewable energy and economies of scale promises to lower costs over time. Furthermore, Ueckerdt et al. (2021) touch upon the importance of incentives and policy support in promoting e-fuel adoption, especially in areas rich in renewable energy resources.^40^ E-fuels can be integrated into existing fuel supply networks and can be operated in internal combustion engines, which may increase their attractiveness as a bridging solution. Current electrolyzer technologies vary in efficiency and cost. Alkaline electrolysis (60–70% efficiency) is mature and affordable but struggles with intermittent renewables due to slow response times. PEM electrolysis (65–75% efficiency) handles renewables better and ramps faster but costs more. Solid oxide (SOEC) offers the highest efficiency (80–90%) by using waste heat, but durability at scale remains unproven. Currently, the production price for e-fuel is somewhat expensive, which is estimated between 2.50 and 4.60 USD per liter in the area of synthetic liquid fuels, as opposed to the 0.40 to 0.70 USD per liter price level for traditional fuels or hydrogen at 0.50 to 1.00 USD per kilo. In 2030, the estimated price decrease due to technology progress will bring the price of e-fuel to 1.40 to 1.60 USD per liter, alongside a parallel decrease in hydrogen as well as synthetic gas fuels. Whereas achieving a worldwide average price of under 2 USD/kg is a pretty long shot, these scenarios have multiple variables to account for, independent of the efficiency provided by the electrolyzers.^8,20^ The main factors mentioned in the scientific literature available include a dramatic reduction in the price of renewables, an acceleration of over 50% in the reduction of the capital price of the electrolyzers, and efficiency improvements.^23^ Furthermore, recent studies have investigated alternative synthesis paths for e-fuels with the aim of increasing efficiency and decreasing hydrogen consumption. For example, methanol-to-jet processes convert e-methanol to e-kerosene more efficiently compared with classical Fischer–Tropsch synthesis. Novel approaches, avoiding hydrogen in the RWGS step by means of solid carbon and the reverse Boudouard reaction and direct electrolysis to e-methanol or hydrogen production by thermochemical water-splitting cycles, seem to have better prospects to reduce energy demand.^8,21^ Figure 1 represents the e-fuel applications.

**Figure 1:**
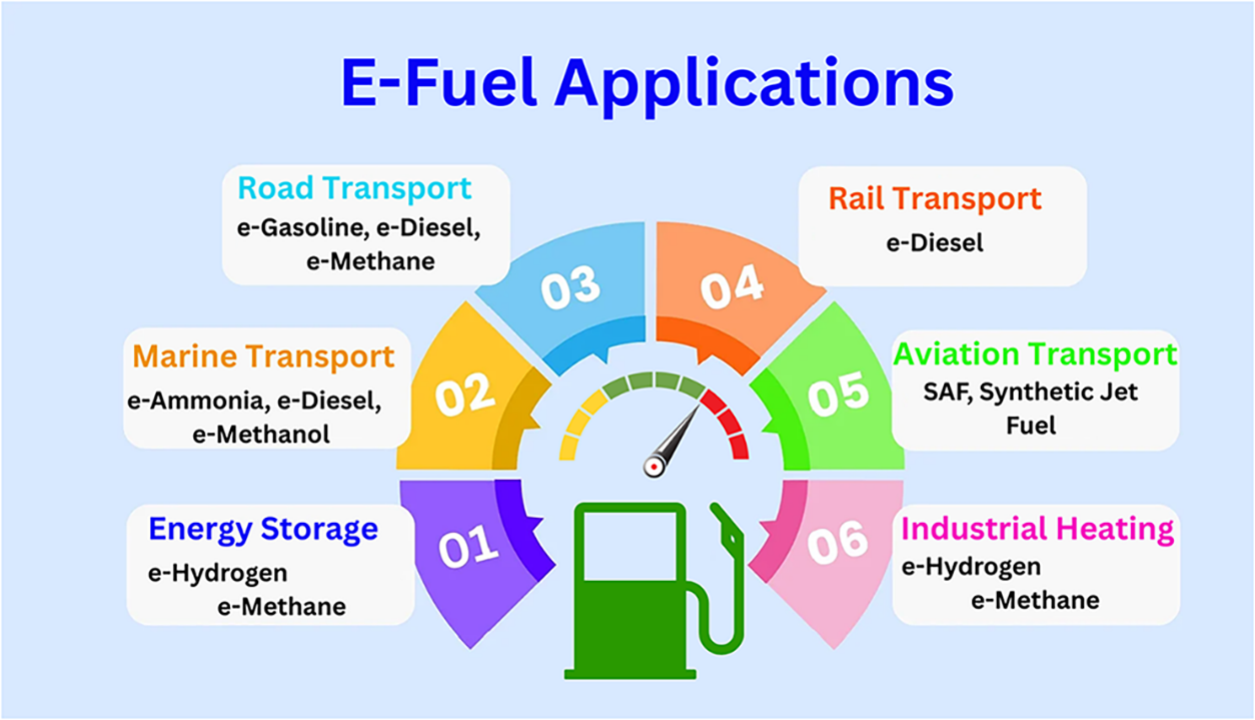
Application of the e-fuel.

One of the most important existing hurdles to the widespread adoption and use of e-fuels is their high production cost, which has been estimated at between three and six US dollars per liter, making them far more expensive than fossil fuels and battery-electric vehicles. Despite future declines in e-fuel costs, these declines are expected to be driven by other factors, such as declining renewable energy costs, widespread adoption, advancements in carbon capture methods, and an overall supportive environment rather than solely by improvements in these e-fuels. This would enable levels of 1.5 US dollars per liter to be reached in medium- to long-term scenarios in specific sectors.^41^ There is still an efficiency gap between BEVs and e-fuels that raises fundamental questions of long-run sustainability. The market potential of e-fuels will ultimately depend on scalability, renewable energy availability, and compatibility with existing fuel infrastructure, while the scale of that market will remain uncertain without significant policy intervention. Empirically effective regulatory instruments to support alternative fuels have included carbon pricing (i.e., the EU Emissions Trading System) and low-carbon fuel mandates (i.e., California’s Low-Carbon Fuel Standard). These policies have effectively narrowed the cost gap of alternatives and encouraged capital investment in the promotion of their use. Subsidies and tax credits, such as those provided in Germany’s PtX development program, have also been critical in scaling e-fuel production. Current energy policies that lean toward electric fuels for aviation and shipping (the EU’s Fit for 55 package) have excluded the role of direct electrification and hydrogen-dominated road and rail.^42,43^ While appreciating these limitations, the research emphasizes that electric fuels can still play a significant role in the decarbonization of hard-to-electrify transport sectors, such as long-haul trucking and long-distance rail.^44^ These electrified fuels can be realized with a combination of technology-neutral carbon pricing, sector-specific mandates, and research and development funding, accompanied by ongoing scaling up of renewable energy generation.^17,45^

## 3 E-Fuels for Road/Railways

In 2023, the worldwide transport sector utilized about 118 EJ (32,778 TWh) of energy, with road transport accounting for 80% and rail for 3%. The industry was emitting 8.7 Gt of CO_2_, which accounted for 23.8% of global emissions.^17,46^ Heavy trucks accounted for almost 40% of road transport emissions, while cars accounted for 55%. If e-fuels become universally adopted, road transport life cycle emissions can be lowered by as much as 85%, recent studies indicate. For instance, using e-diesel instead of traditional diesel in heavy trucks would reduce emissions by 78% per vehicle yearly. In the case of cars, the adoption of e-gasoline would bring down emissions by 65% per vehicle yearly.^10^ Road transport requires high-energy-density fuels owing to operational conditions. E-diesel and e-gasoline with energy densities of 38.6 and 34.2 MJ/L, respectively, perform the same as fossil-derived fuels. Road transport in Europe utilized 275 Mtoe (3196 TWh) in 2022, recovering after the COVID-19 pandemic. If 50% of this demand were fulfilled through e-fuels, CO_2_ emissions would decrease by 350 Mt per year. Additionally, e-fuels can contribute to energy security through decreased fossil-fuel imports. Figure 2 represents the global CO_2_ emissions trends (2010–2025).^47–49^

**Figure 2:**
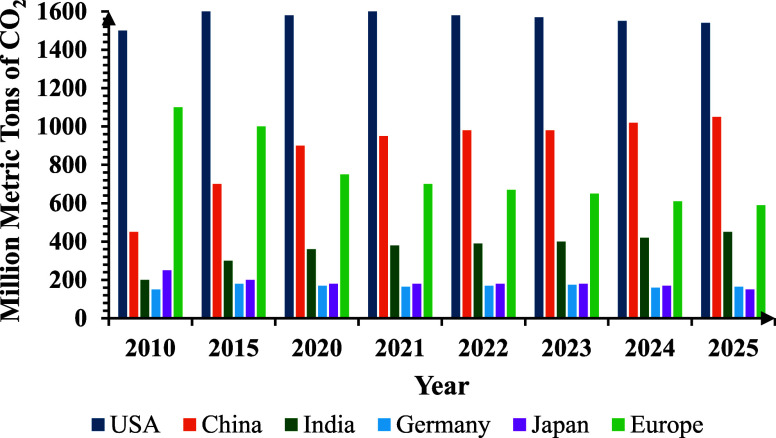
Global CO_2_ emission trends (2010–2025).

Furthermore, the following points provide a perspective on the impact of the current tax treatment of e-fuels in Germany’s energy and transportation sectors.^47^ E-fuels are marketed as a bridge technology for industries moving toward electrification at a relatively fast pace, such as road transport and light- and medium-duty vehicles. On the other hand, in hard-to-abate sectors such as long-haul air transport, maritime transport, and heavy freight transport, where direct electrification remains technically and economically challenging, the only feasible long-term strategy appears to be e-fuels.^49^ Figure 3 represents the illustrative global CO_2_ emission trends by transport sector (2015 to 2025).

**Figure 3:**
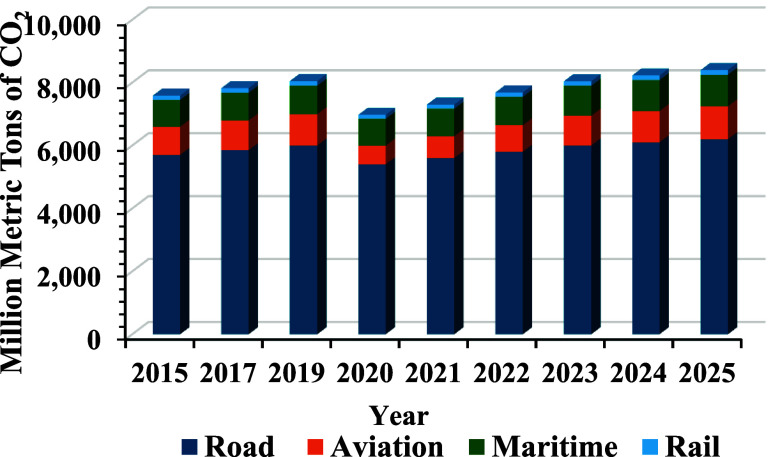
Illustrative global CO_2_ emission trends by the transport sector (2015–2025).

Nikolina’s study, described by the International Renewable Energy Agency (IRENA),^48^ approximates that by 2035, e-fuels can provide up to 20% of road transport energy demand in developed economies and reduce CO_2_ emissions by 1.4 Gt per year. For rail transport, e-diesel provides a promising alternative, where electrification is not economically or technically viable. Powering railway networks may involve a significant investment in infrastructure,^49^ which might be impractical for rural or developing areas.^50^ E-diesel is a realistic option for decarbonizing rail transport where it is not possible or economically justified to fully electrify.^51^ Garcia’s studies emphasize compatibility with existing fuel infrastructure, and decarbonizing e-diesel also represents a step toward long-term carbon neutrality. From the Ahsan studies, depending strongly on the CO_2_ sourcing pathway and electricity mix, e-diesel can achieve up to ∼70% life cycle GHG emission reductions compared with fossil diesel. Moreover, by offering a substitute for fossil diesel, it enables ongoing utilization of internal combustion engine assets while removing the carbon and financial costs of any new infrastructure.^52^ Eighteen percent of rail lines globally are still diesel powered and consume around 52 TWh of energy. Studies estimate that replacing fossil diesel with e-diesel by half, giving a lower bound estimate of 50%, of these diesel lines could save around 25 Mt CO_2_ a year.^53^ In India, strategically using e-diesel on nonelectrified routes has the potential to reduce railway emissions by as much as 40% and further substantiate the need for government support for e-diesel policy development and market scale-up.^54^ Figure 4 presents the comprehensive e-fuel production routes.

**Figure 4:**
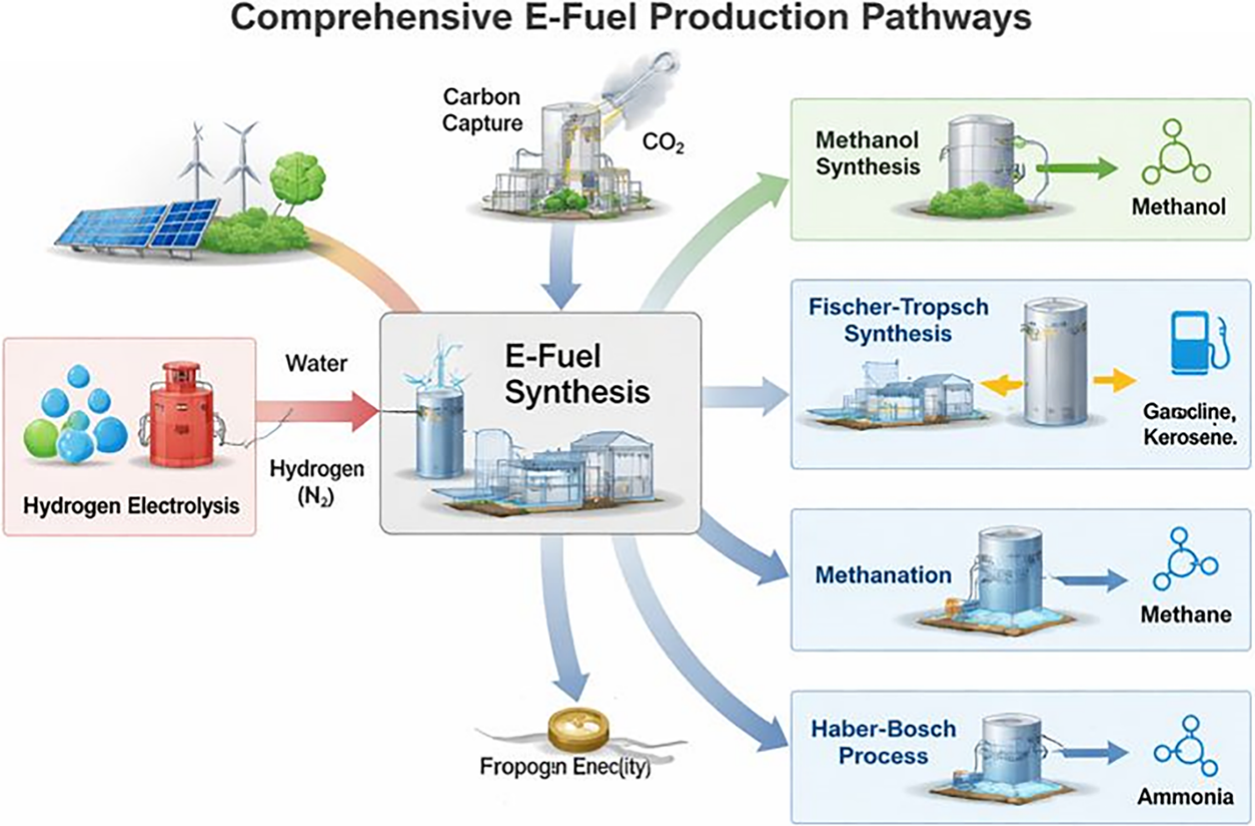
Comprehensive e-fuel production pathways.^25^

The most often cited 70–90% GHG emission reduction potential of e-fuels is indeed representative of a rather broad range, which is utterly reliant on the chosen CO_2_ sourcing mechanism because of significant differences in their energy intensities considered.^55,56^ E-fuels, in contrast, using lower-energy carbon sources such as biogenic carbon capture or flue gas capture in an industrial context, could come rather close to achieving the high end of this range, approaching 90% reductions using renewable power.^4^ Instead, the amount produced through energy-intensive direct air capture (DAC), requiring 1.0 to 2.5 MWh CO2/ton CO_2_, will typically be well below the 70% cut, often at or below 70%. This explains the significant difference in the emissions disadvantage of the DAC-based e-fuel in comparative analyses, while the e-fuel generated from optimal carbon sources is reportedly capable of providing deeper decarbonization than the BEVs with superior efficiency due to the extensive demand for renewable electricity per kilometer.

### 3.1 Significance of E-Fuel Adoption for Road and Rail

- E-fuels can be easily used in today’s ICE-fueled vehicles with minimal modifications, opening the way to a seamless transition to cleaner energy.^48^
- E-fuels, produced using captured CO_2_ and renewably generated energy, reduce net CO_2_ emissions compared to gasoline and diesel. This significantly reduces greenhouse gas emissions.^4^
- Complementary to current gas stations, pipeline networks, and distribution systems, e-fuels avoid the massive investments in developing new charging or hydrogen refueling infrastructure.^54^
- Well-suited for long-haul trucks, freight trains, and rural transportation where battery electrification is not feasible because of range, weight, and infrastructure limitations.^17^
- E-fuels emit fewer pollutants than fossil fuels, lowering nitrogen oxides (NOx) and particulate matter emissions, which are detrimental to public health and the environment.^2^
- E-fuels also show an overall reduction of tailpipe emissions compared to conventional gasoline and diesel fuels; considering the complete life cycle, however, some aspects are potentially higher than those of their fossil-fuel counterparts, for example, e-diesel^3^ or e-gasoline,^9^ suggesting a holistic assessment of environmental impact.^2^
- Countries can decrease their reliance on imported fossil fuels by locally producing e-fuels from renewable sources, improving energy security and resilience.^53^
- E-fuels trap excess renewable energy in liquid form to keep excess electricity generated by solar and wind power captive as transportable fuel, so it cannot be wasted.^53^
- Production of e-fuel creates employment in fuel technology, manufacturing, and renewable energy sectors, stimulating industrial and economic development.^46^
- By stopping deforestation, lowering oil drilling pollution, and cutting carbon emissions, e-fuels make world climate action and sustainability possible.^51^
- E-fuels can be used anywhere in the world, including regions that have no EV or hydrogen infrastructure, and thus, they offer a vital solution to address global climate targets like the Paris Agreement.^48^

### 3.2 Challenges in E-Fuel Adoption for Road and Rail

- E-fuels are priced at 3–6 USD per liter, which is less competitive than electric power and traditional fuels.^57^
- Reduce the price below 1.50 USD per liter (by 2030) to achieve more widespread usage.^58^
- Well-to-wheel efficiency is merely 10–20%, versus 70–80% with BEVs.^57^
- Major energy losses occur during e-fuel production, conversion, and combustion.
- Current fuel stations will have to be modified to accept e-fuels, requiring additional investment.^42^
- There are limited large-scale manufacturing facilities operating, limiting supply.^43^
- Requires massive amounts of green electricity to be meaningfully carbon neutral.^17^
- Direct electrification competition (BEVs, rail infrastructure) for renewable supply of energy.
- Needs CCU technology with high efficiency.^45^
- Technical difficulties and prohibitive expense of large-scale CO_2_ capture.^53^
- BEVs and HFCVs are more efficient and have more favorable policy incentives.^25^
- Electric train systems are increasing, lowering the demand for e-fuels for transportation by train.
- E-fuels cut CO_2_ but still emit NOx and particulate emissions upon combustion.^2^
- Not an entirely zero-emission option, such as BEVs or hydrogen.^17^
- Governments center more on e-fuel subsidies and hydrogen installations, with questionable regulatory backing for e-fuels.^26^
- Lack of long-term incentives deters large-scale investment in e-fuel technology.^17^

## 4 E-Fuels for Marine/Waterways

The shipping sector, accounting for about 3% of the world’s CO_2_ emissions, is investigating e-fuels as a promising route to decarbonization.^59^ E-fuels produced from renewable electricity are a potential alternative to conventional marine fuels. E-fuels like e-methanol and e-ammonia are made by mixing hydrogen derived from renewable energy-driven electrolysis with sequestered carbon dioxide or nitrogen.^60^ These can significantly reduce greenhouse gas emissions when used to replace conventional marine fuels. Regional feasibility is important for determining low-carbon transport and is particularly relevant for developing economies with limited grid reliability and renewable energy capacity. In these circumstances, deployment of battery-electric vehicles (BEVs)^61^ is doubly problematic, given the instability in electricity supply and lack of charging infrastructure. Hydrogen fuel cell vehicles (HFCVs) will also require investment in hydrogen production facilities and hydrogen distribution networks, which do not lend themselves easily to short-term feasibility.^62^ Conversely, e-fuels represent a practical solution to support economies with an existing internal combustion engine (ICE) infrastructure and distribution systems. It has been suggested that e-fuels can also help decarbonize areas with limited electrification by using imported or regionally produced synthetic fuels, although e-fuels are fairly expensive to produce and require renewable energy inputs, which, without supportive policy and investment, will not make them feasible. Figure 5 represents the challenges of e-fuel for the marine system.

**Figure 5:**
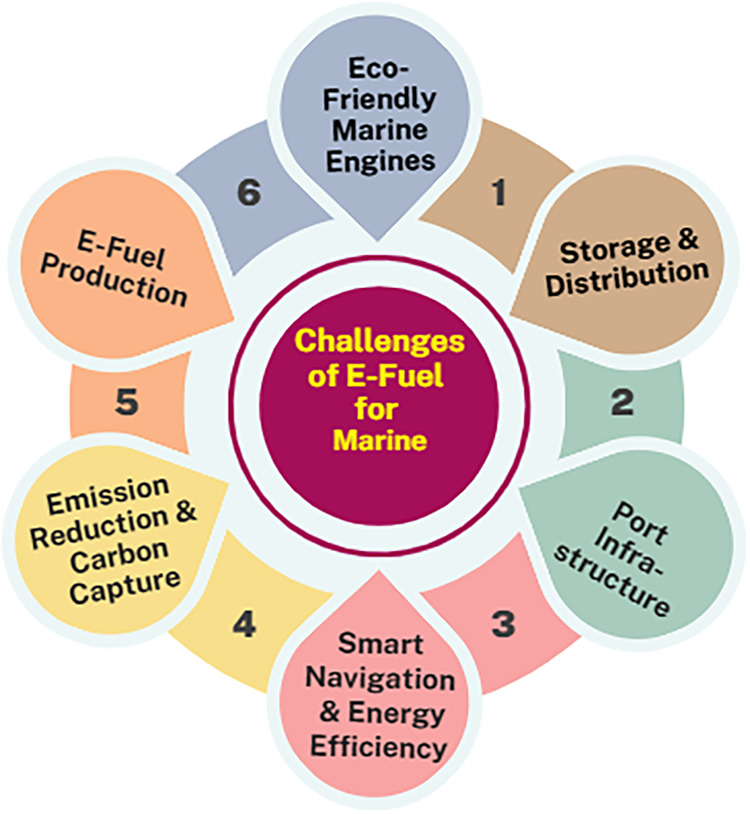
Challenges of the e-fuel marine system.^59^

Maersk in 2024 introduced the Alette Maersk, the world’s first low-carbon methanol-powered container ship, marking a strategic technological turning point in the decarbonization of maritime transportation and complementing the company’s net-zero ambition for 2040 despite the sparse refueling network.^63^ Currently, nonconventional fuels account for only about 3% of Maersk’s 10–11 million metric tons of fuel purchased each year, but the company plans to expand this to 15–20% by 2030.^64^ Complementing such efforts, the Zero Emission Maritime Buyers Alliance (ZEMBA), founded by Amazon, IKEA, and others, is accelerating the commercial viability of near-zero-emission e-fuels. Their first procurement contract, awarded to Hapag-Lloyd, involves the use of biomethane projected to reduce emissions by approximately 82,000 tonnes over two years. Despite such efforts, e-fuels remain more than double the price of traditional marine fuels owing to high-energy production pathways, feedstock costs, and supply constraints.^65^ Recent techno-economic research highlights the importance of reducing costs through advancements in electrolyzer efficiency, the integration of carbon capture technologies, and the scale-up of synthetic fuel synthesis.^66^ There will be significant capital investment in infrastructure, including green hydrogen production plants, liquefaction terminals, and multinational bunkering systems. To assist in this, actors across the supply chain, such as trade associations and technology, finance, and energy innovators, are advocating for carbon pricing mechanisms and subsidies to help level the playing field. Ongoing EU and IMO pilot programs, such as the FuelEU Maritime initiative, demonstrate how the dual frameworks of policy and public-private cooperation are increasingly driving R&D initiatives and market readiness. E-fuels can revolutionize decarbonization for deep-sea shipping, but this requires parallel pathways of technological advancement, regulatory policy alignment, and financial instruments to decouple costs from fossil-fuel alternatives.^67^

A review of several studies was conducted to examine the comparison, impact, and advancements of marine fuels.^65,67–76^ Emissions from maritime transport can be reduced by up to 30% through optimization, slow steaming, and energy-saving technologies. Scrubbers are used to lower SO_*x*_ emissions from heavy fuel oil, while LNG can reduce CO_2_ emissions by 20–25% compared to marine diesel.^67^ However, LNG carries the risk of methane slip, a gas with over 80 times the global warming potential of CO_2_ over 20 years, and its global refueling infrastructure remains limited.^74^ Biofuels and synthetic fuels can achieve 50–80% lower life cycle emissions but face drawbacks such as 2–5× higher costs, sustainability challenges, and limited supplies. E-fuels are 3 to 5 times more costly than traditional marine fuels, requiring subsidies in order to be competitive.^77^ Additionally, CO_2_ capture systems onboard ships offer a promising solution to cut carbon emissions. These systems, typically using amine-based chemical absorption, can remove 85 to 90% of CO_2_ from exhaust gases. The captured CO_2_ is compressed, liquefied, and stored onboard for offloading at the port. However, these systems require up to 25% of engine power for operation, impose significant space and weight demands, and depend on port infrastructure for CO_2_ handling.^75^ If powered by nonrenewable sources, the overall emission reduction benefit is substantially reduced. Electric fuels provide a much-needed pathway to a clean sea transport system, as well as an inland waterway system.^74,75^ Conventional fuels, including heavy fuel oil (HFO) and marine diesel oil (MDO), are identified to be highly carbon-emitting, while electric fuels, such as e-diesel, e-gasoline, e-gas, and e-hydrogen, with assistance from ammonia (NH_3_), are well identified to significantly reduce CO_2_ emissions, NO_*x*_, SO_*x*_, as well as particulate matter by a considerably higher percentage compared to other sources.^76^ Table 2 represents a comparison study of marine fuel.

**Table 2:** Comparison of Marine Fuels Impact and Advancements

**fuel**	**pollutants**	**water risk**	**CO**_**2**_ **impact** (g/MJ)	**innovations/technology**	**adoption** (%)
HFO^67^	high SO_*x*_/NO_*x*_/PM	toxic spills	3.1–3.5	scrubbers (85% of SO_*x*_)	62
MDO^65,67–76^	lower SO_*x*_, high NO_*x*_	moderate spills	2.8–3.0	SCR (90% of NO_*x*_)	60
LNG^74^	low SO_*x*_, but CH_4_ slip	methane leaks	25% CO_2_ (GWP 36×)	dual-fuel engines	69
e-diesel^75^	near-zero pollutants	biodegradable	0.05 (wind-PtL)	PtL (70% eff.) + Haru Oni plant	70
e-hydrogen^65,67–76^	low CO_2_/PM (H_2_O only)	no spills	0.1–0.2 (depending on electricity source)	PEM electrolysis (80% eff.)	71
ammonia^63^	low CO_2_, but high NO_*x*_	toxic risk	0.1–0.3 (green H_2_ pathway)	renewable H_2_ (60% eff.)	65

### 4.1 Significance of E-Fuel Adoption for Marine/Waterways

- Enables deep-sea decarbonization.^25^
- “Drop-in” compatibility with existing ships.^51^
- Near-zero emissions from the well to the wake.^74^
- Ensures compliance with IMO and EU regulations.^75^
- Boosts energy security and renewable integration.^2^

### 4.2 Challenges in E-Fuel Adoption for Marine/Waterways

- Most maritime players do not know that e-fuels can work or doubt their viability, hindering adoption in the sector.^65^
- Most ports do not have e-fuel storage, bunkering, and distribution infrastructure, necessitating heavy investment.^75^
- E-fuels need large amounts of renewable energy, often unavailable in remote or developing regions.
- Meeting global shipping demand (over 300 million tons of fuel annually) is a huge challenge.^57^
- No clear international standards or policies for e-fuels in maritime use create uncertainty.
- Conventional fuels like HFO remain cheaper and more accessible, slowing e-fuel adoption.
- Electrolyzers and carbon capture tech are still developing, limiting e-fuel production efficiency.^43^
- E-fuels’ environmental benefits vary based on renewable energy sources and carbon capture methods.^25^
- International shipping requires unified standards, infrastructures, and policies, which are complex to establish.^35^
- Many in the maritime industry are unaware or skeptical of e-fuels, slowing their adoption.

## 5 E-Fuels for Aviation

The aviation sector is a significant contributor to greenhouse gas emissions worldwide, accounting for 2.5% of total CO_2_ emissions and roughly 2% of emissions related to energy.^78^ With the demand for air travel predicted to rise by 3–4% annually, the industry is facing mounting pressure to replace conventional jet fuel with sustainable alternatives.^79^ E-fuels are a promising option, with the potential to decarbonize air travel without requiring significant modifications to current aircraft or infrastructure.

Sustainable aviation fuels (SAFs), as defined in the literature, include both biobased fuels and synthetic fuels (e-fuels) such as e-kerosene, provided they meet established sustainability criteria. Therefore, e-kerosene is also considered an SAF rather than a separate category. Currently, global SAF production, including both biobased and synthetic variants, amounts to approximately 1 million tons, which falls short of the projected 1.5 million tons.^80,81^ SAFs account for about 0.3% of jet fuel consumption worldwide, with projections indicating a modest increase to 0.7% by 2025. This highlights a significant gap between current production capacity and the scale required to meet aviation’s fuel demands sustainably.^82^ For context, global jet fuel consumption in 2022 was around 300 million tons, emphasizing the enormous scale-up in SAF production, including e-fuels, needed to make a meaningful impact.^21^ Figure 6 represents the five pillars of E-fuels for aviation.

**Figure 6:**
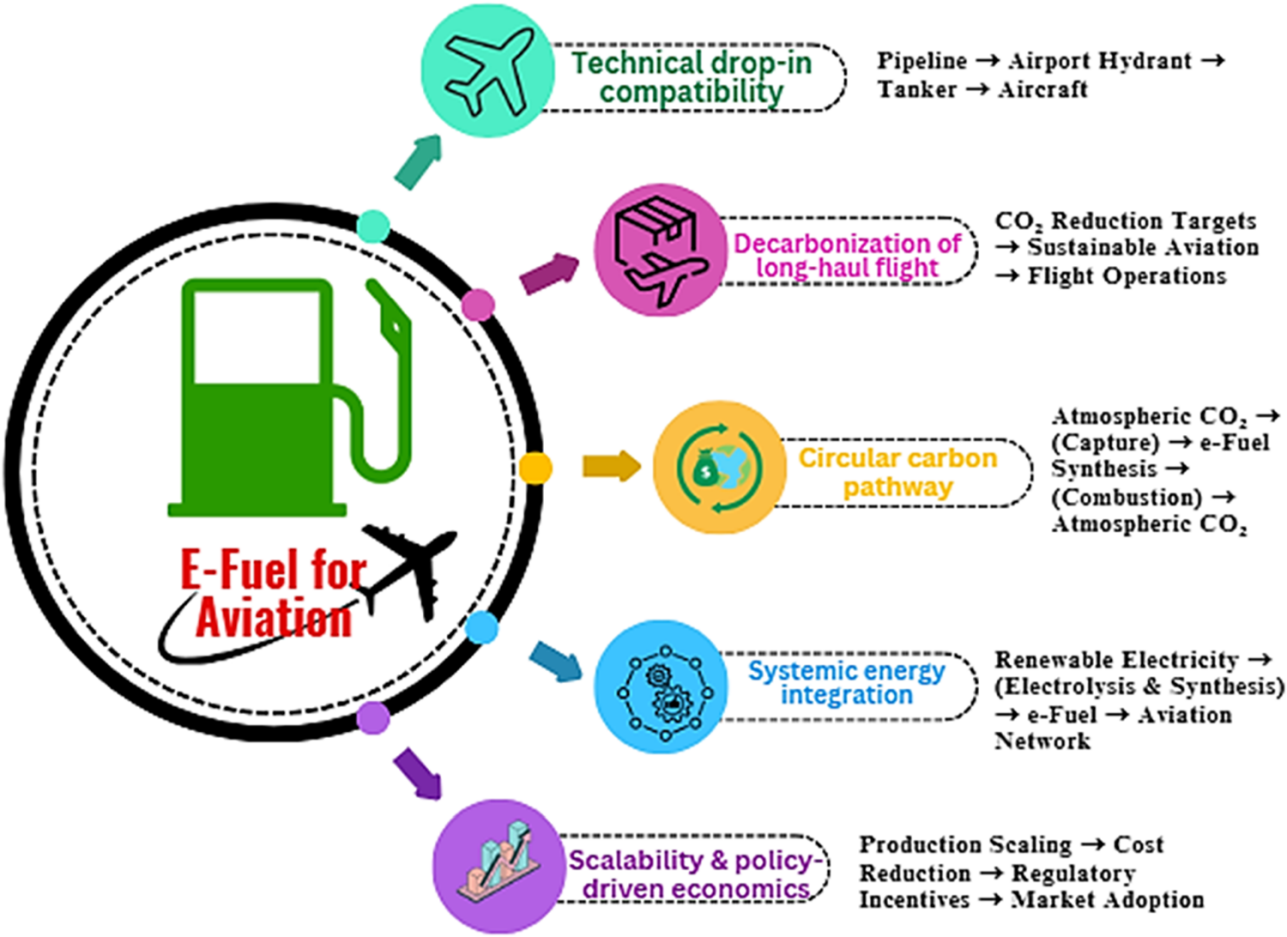
Five pillars of e-fuel for aviation.^80^

To bridge this deficit, extensive investment and policy actions are already underway. According to de Oliveira’s study, the global capacity of SAF is nearly 6 billion gallons in 2030, a 4-fold increase from 1.3 billion gallons in 2023.^83^ For instance, the Minnesota-based Pine Bend Refinery is targeting production of up to 1 billion gallons of SAF per year by 2025. Yet, the sustainability of such initiatives largely relies on supportive financial models, including tax credits and loans, which are vulnerable to political changes. According to the European Union’s “Refuel EU Aviation” project, it is estimated that 2% of aviation fuel will be sustainable by 2025, 5% by 2030, and 63% by 2050.^84,85^ These goals, in conjunction with the reduced prices of renewable energies and technological advancements, are anticipated to lower the production costs of e-fuels from the current 3–6 EUR per liter to around 1.5–2.5 EUR per liter by the year 2030. However, this particular range is a conditional estimate, with high dependence on the assumed scenario, in which case the costs are based on the assumption of reduced renewable electricity costs of 30–50 EUR/MWh, learning curves of up to 60–70% cost reduction in electrolyze capital costs, and the use of high carbon pricing (100–150 EUR/tCO2). Hence, the lower end of this cost range, 1.5 EUR per liter, may be characterized by an optimistic scenario where all three wishful thoughts could simultaneously turn true, while higher costs of electricity, reduced learning curves for electrolyzers, and reduced carbon policies could move the costs to the upper end of this range and even above. The shift to e-fuels, however, is challenged by major economic and policy hurdles.^4,32^ There is also a mismatch between supply and demand: airlines are unwilling to buy e-fuels unless there is stable pricing and adequate supply, while producers are unwilling to expand production unless there are assured long-term orders. Alongside this, the promise of e-fuels is particularly policy-sensitive as financing incentives may change with shifts in administration. However, the Barton et al.’s study suggests that e-fuels have strong potential to decarbonize aviation.^86^ With investment, technological advancement, and robust policy support, e-fuels might be instrumental in achieving the air transport sector’s net-zero carbon emissions aspirations by 2050.

Biobased fuels hybrid routes, combined with power-to-liquid (PtL) e-fuels, are seen as the sensible thinking-ahead roadmap for aviation decarbonization in the context of actual resource constraints.^87^ This process of hybridization makes it feasible to partly substitute the jet fuel produced from fossil sources and thereby reduce the challenges posed by the sustainability biomass availability constraint and the high electricity demand in the production of fully synthetic PtL fuels.^88^ By mixing bio-SAF with PtL fuels, supply chain reliability can be strengthened while taking advantage of existing fuel production and distribution infrastructures with minimal modification. Nevertheless, their usage is faced with a number of challenges, such as increased production costs compared to traditional jet fuel, variability in feedstock supplies, competition for renewable electricity, and sustainability reporting.^89^ In spite of these limitations, hybrid SAF routes provide a scalable transition tool that may help to accelerate the adoption of SAF in the short term and facilitate a gradual transition to fully synthetic and advanced biofuel sources. Table 3 represents the comparison of aviation fuels.

**Table 3:** Comparison of Aviation Fuels

**aspect**	**e-fuels** (PtL)	**conventional jet fuel**	**biofuels** (SAF)	**trend-based insight**
feedstock^86^	renewable electricity, H_2_O, CO_2_	fossil crude	biomass (algae, waste)	CO_2_-based synthesis promotes circular carbon economy
process^87^	electrolysis + Fischer–Tropsch	refining & cracking	hydroprocessing, fermentation	e-fuels evolving; energy intensive
emissions^88^	reduce up to 90% (renewable-based)	100% CO_2_ baseline	50–80% reduction (feedstock)	biofuels + CCS may achieve lowest life cycle GHGs
output (2023–2024)^89^	∼50 kt/year	∼300 Mt/year	∼1 Mt/year	e-fuels <0.02% of demand; scale-up critical
energy density^86^	comparable to jet A	high	high	compatible with existing engines
infrastructure^90^	new PtL plants needed	fully deployed	retrofit/new biorefineries	SAF more flexible; PtL faces barriers
scalability^90^	high (green H_2_/CO_2_-limited)	resource-constrained	biomass-constrained	PtL depends on renewable deployment
policy support^86,87^	strong (EU Fit for 55, SAF mandates)	declining	moderate (blending targets)	policies favor e-fuels & SAFs
environmental impact^4^	near-zero (renewable-powered)	high GHGs & pollutants	moderate; land-use concern	e-fuels minimal footprint if green-powered
adoption^91^	pilot flights (Lufthansa, Airbus)	universal	expanding (United, KLM, etc.)	SAF adoption leads; PtL gaining traction
challenges^88,89^	high CapEx, green H_2_-dependency	emission-heavy, finite	biomass availability, land-use	PtL scale-up and cost reduction needed

Cabrera believed that, made using renewable electricity, water (through electrolysis), and CO_2_ (extracted from the atmosphere or industrial emitters), e-fuels have the potential to reduce CO_2_ by as much as 90% relative to fossil jet fuel.^87^ However, their widespread adoption is reportedly hampered by high energy losses (40–55%), restricted renewable energy supplies, and high production prices (3–6 EUR/l compared with 1–2 EUR for bio-SAF and 0.5–0.7 EUR for fossil jet fuel). However, other studies have reported that their mass adoption comes at the cost of high energy costs (40–55% efficiency losses), scarce renewable energy resources, and expensive production.^32,88^ Despite these hurdles, the deep policy commitment of the EU’s RefuelEU Aviation mandate and ICAO’s CORSIA mechanism is driving investment in PtL technology. Airlines such as Lufthansa and Airbus have started testing 100% e-fuel-powered flights, proving feasibility but pointing to the imperative of scalability and cost reduction.^91^ To achieve maximum impact, various studies are focusing on electrolysis efficiency improvements, large-scale carbon capture integration, and hybrid SAF solutions incorporating biofuels and electric fuels to establish a cost-effective and sustainable decarbonization pathway for aviation.^92,93^

Zamboni et al. describe a well-to-wake (WTW) emissions framework that evaluates life cycle greenhouse gas (GHG) emissions from fuel feedstock production and processing (well-to-tank, WTT) through aircraft engine combustion (tank-to-wake, TTW).^94^ Unlike traditional TTW analyses, which consider only in-flight emissions, WTW accounting incorporates upstream energy use, refining, transport, and end-use combustion, providing a more comprehensive estimate of climate impact.^95^ In this study, emission reductions for sustainable aviation fuels (SAF) are therefore discussed on a WTW basis, unless otherwise stated; under this boundary, HEFA-based SAF achieves 50–80% lower emissions, while power-to-liquid (PtL) e-fuels can achieve up to 90% CO_2_ reduction when produced using renewable electricity. Conventional fossil jet fuel gives ∼80–100 gCO_2_/MJ and no net carbon gain.^96,97^ Yet feedstock constraints, land-use change, and the high-energy-intensive synthesis of the fuel affect the actual-world sustainability of SAF. Policymakers and industry leaders employ WTW metrics to enhance aviation decarbonization strategies, maximize SAF blending requirements, and analyze the long-term sustainability of reducing net CO_2_ emissions from flights within international climate goals such as Net Zero 2050.^98^

Several countries have led the way with long-term incentive frameworks to scale e-fuels, providing models to replicate. The EU requires e-fuel quotas (e.g., ReFuelEU Aviation) and combines them with carbon pricing under its Emissions Trading System (ETS). Germany’s H2Global program employs competitive auctions and contracts for difference (CfDs) to derisk investments. The US uses tax credits (3USD/kg green H_2_ under the IRA) and state-level low-carbon fuel standards (e.g., California’s LCFS). Norway blends carbon taxation and air fuel quotas, whereas Chile attracts investment through subsidies for renewable energy. These models demonstrate the success of hybrid policies (mandates, subsidies, and carbon pricing) specifically designed to align with local resources and industries.

### 5.1 Significance of E-Fuel Adoption for Aviation

- Produced from renewable power and CO_2_ captured from the air, e-fuels reduce aviation emissions by 90%.^35^
- Reduces contrails, particulate matter, and NOx, decreasing aviation’s overall climate impact footprint.^95^
- Can operate on current aircraft engines without any changes.
- Compatible with existing fuel storage, transportation, and refueling infrastructure.
- Suited for long-haul flights where batteries and hydrogen are not sufficient.
- Makes reducing net CO_2_ emissions from transoceanic flights possible, the biggest source of emissions from aviation.^22^
- Assists airlines in meeting CORSIA and net-zero targets by 2050.^84^
- It can supply global jet fuel needs (more than 300 million tons per year). Transforms surplus renewable energy into liquid aviation fuel.^87^
- Supported by EU’s “ReFuelEU Aviation” and airlines such as Lufthansa, spurring adoption and innovation.^41^

### 5.2 Challenges in E-Fuel Adoption for Aviation

- E-fuels are 3 to 5 times more expensive than conventional jet fuel, requiring subsidies to be viable.
- Large amounts of renewable energy are needed for e-fuel production, straining energy grids.^17^
- Airports lack e-fuel storage, distribution, and refueling systems, needing significant investment.^87^
- Meeting global aviation fuel demand (over 300 million tons annually) is a major challenge.^85^
- Lack of clear international standards and policies for e-fuels creates uncertainty for airlines.^32^
- Conventional jet fuel remains cheaper and more accessible, slowing e-fuel adoption.^4^
- Electrolyzers and carbon capture tech are still developing, limiting e-fuel production efficiency.^32^
- E-fuels’ environmental benefits depend on renewable energy sources and carbon capture methods.
- International aviation requires unified standards, infrastructure, and policies, which are complex to establish.^88^
- Many in the aviation industry are unaware or skeptical of e-fuels, slowing their adoption.^87^

Hybrid systems that pair battery-electric (BEV) technology with e-fuel combustion engines are coming under the spotlight for areas such as shipping and aviation, where complete electrification is still problematic.^99^ In aviation, hybrid-electric plane concepts can use batteries for takeoff and landing and e-fuels for cruising, maximizing energy efficiency, and reducing emissions. In maritime applications, hybrid drive systems allow for short-distance travel on electric power and long-distance voyages on e-fuels. Research indicates that these systems can reduce GHG emissions by 30–50% compared to conventional fuels, though bioderived fuels with CCS may achieve even lower life cycle emissions. Sustainability is, however, contingent on innovation in terms of energy density, storage means, and enabling infrastructure.^100^ Furthermore, e-fuels deliver better performance in reducing non-CO_2_ emissions than traditional fuels across all transport sectors. The evidence indicates:

- NOx: 30–50% lower for marine/shipping use.
- SOx: Virtually zero emissions owing to sulfur-free production.
- Particulates: 70–90% cutting of aviation.

Yet combustion conditions and engine architecture have a strong bearing on outcomes. While e-fuels remove sulfur and reduce particulates by half, reductions in NOx rely on combustion optimization, with fuel cells performing better than ICEs. Life cycle air quality effects of mass production remain an area of research gap. Regulatory policies (e.g., IMO 2020 sulfur limit) amplify these advantages, making e-fuels key to multipollutant mitigation in hard-to-electrify domains.

## 6 Techno-Economic Feasibility and Market Barriers of E-Fuels

The techno-economic feasibility of e-fuels will depend on three critical factors: (i) affordability and access to renewable energy, (ii) maintaining trends in efficiencies for electrolyzers and for carbon capture/CO_2_ utilization (CCU), and (iii) being competitive with fossil-fuel prices.^101^ While there has been some development around electrolyzer design and together with the integrated CCU systems,^24^ the production costs are still well beyond the realm of feasibility (3.50–7.00 USD/L vs 0.50–1.00 USD/L for conventional fuels^32^) due to the significant energy demands and immature supply chain.^102,103^ At present, there are a number of European Union and national developments (such as the projects at the level of a Green Deal) seeking to demonstrate a path to scalable production, and while this is a welcome development, the path to commercialization will take time due to persistent obstacles such as inconsistent policy frameworks (only 10% of countries have clear biofuel mandates) and underinvestment (30% less total funding than other energy sectors).^104^ Across the board, we need to see significant developments in our renewable energy costs, electrolyzer and CCU performance, and even creative modes of financing (e.g., carbon pricing, subsidies) to close the current 5–10× price point gap. Without cohesive and coordinated policy, industry, and R&D efforts, e-fuels will remain a niche solution avoided by greening industries; they need to become a mainstream decarbonization pathway.^104,105^ A wide roll-out of e-fuel appears to be easier in certain regions than in others because of differences in renewable resource endowment and land area constraints. A country with an established grid and low-cost, available renewable sources would be better off in terms of combining various power sources for e-fuel, while a nation with a less stable grid and relatively expensive sources of electricity or limited land for implementing solar and wind sources would be presented with hurdles.^40^ Regional differences indicate that the adoption of e-fuel solutions will happen in different ways in different places across the world and that there is a need for realistic policy support based on regional considerations. Infrastructure constraints are major technical constraints for the implementation of alternative fuels.^8^ Handling ammonia as a bunkering fuel involves careful consideration of material compatibility and resistance to corrosion, as well as specialized safety measures, because it is a toxic fuel. Implementation of e-methanol as a fuel requires adjustments in storage tanks, gaskets, and fuel-handling systems because of differences in chemical composition and lower flash point values compared to conventional fuels.^67^ Similarly, hydrogen as a fuel has critical safety challenges with respect to hydrogen embrittlement in storage and transport infrastructure, which demands the use of special materials and safety standards. Figure 7 represents the e-fuel adoption barriers.

**Figure 7:**
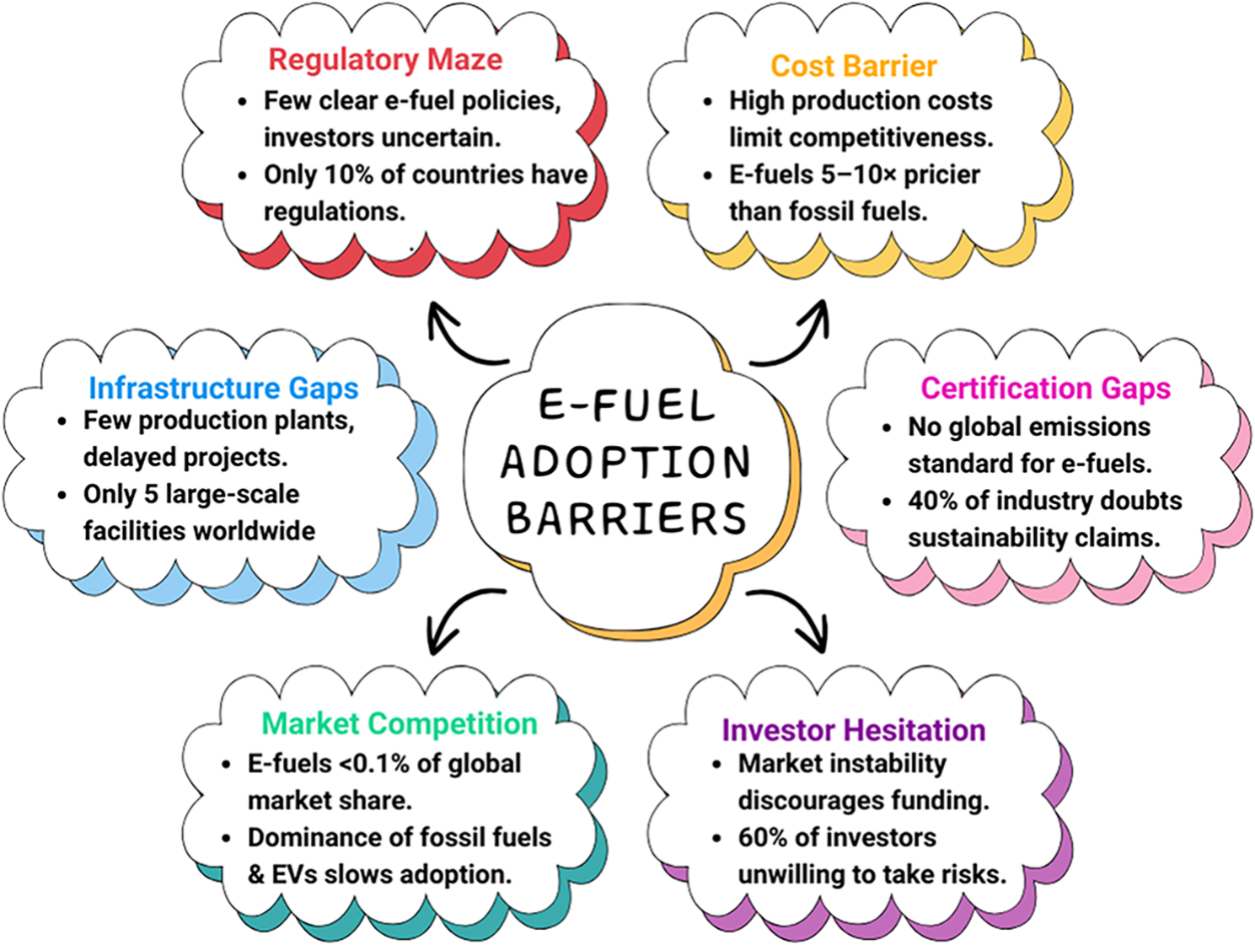
E-fuel adoption barriers.^24^

Scaling e-fuel production faces several critical logistical bottlenecks, primarily the limited availability of low-cost renewable electricity, carbon dioxide sourcing, and infrastructure for large-scale synthesis and distribution.^106^ The production of e-fuels poses a significant risk of burden shifting, in which emission reductions in the transportation sector may be offset by increased environmental pressures elsewhere in the value chain. Specifically, the substantial renewable electricity demand required for electrolysis and fuel synthesis can induce secondary impacts related to water consumption, land-use change, and ecosystem degradation if energy systems remain reliant on resource-intensive technologies. Without proactive mitigation strategies, these effects may compromise the net environmental advantage of e-fuel deployment. Consequently, a comprehensive life cycle sustainability assessment encompassing not only carbon accounting but also water footprint, land-use intensity, and biodiversity implications is essential to validate the holistic environmental impact of e-fuel systems.^4^

To generate 1 L of e-fuel, approximately 5–7 kWh of renewable electricity is required, underscoring the urgent need to increase clean power capacity globally. Carbon capture technologies like direct air capture (DAC) are extremely energy-intensive, with 1.0–2.5 MWh of energy consumed to capture just 1 ton of CO_2_, restricting scalability absent dramatic improvements in efficiency.^23^ Necessary upgrades to distribution for fuels also exist, with projected costs of approximately 10 USD EUR billion worldwide to develop infrastructure at scale for synthetic fuels.^107^ A supply chain optimization study has recommended, creating localized production hubs near renewable energy and carbon source locations to also limit transportation-related emissions, reducing them by as much as 30% and the overall supply chain cost by as much as 15–20%, and thereby improving overall supply chain viability.^108^ Currently, only a few large-scale e-fuel production facilities exist globally, which are unlikely to meet the demand potential, resulting in delays and stalling of approximately 80% of prospective e-fuel projects due to infrastructure constraints. Currently, e-fuels hold less than 0.1% of the global fuel market share and add to the market uncertainty and competitive pressures from lower-cost fossil fuels and BEVs, striking investor confidence. Furthermore, retrofitting fuel stations for e-fuels costs 500 K to 1 M USD per station (storage, pumps, safety upgrades), while port bunkering terminals require 10 to 50 M USD per facility due to scale and regulations. Because of this, global infrastructure upgrades could exceed 20B USD. By focusing on upgrades in high-demand areas and progressively rolling them out, improvements can be made to costs and the timeliness of e-fuel uptake.

Surveys show consumer awareness at moderate levels but with growing interest in industries where electrification is not feasible. A 2022 Eurobarometer survey found that only 28% of the public had heard of electric fuels, but more than 60% supported alternative low-carbon aviation and shipping fuels.^109^ Industry players, particularly in aviation and heavy transport, are showing strong readiness, citing infrastructure compatibility as a key benefit. But doubts about regulatory support and long-term cost competitiveness are softening enthusiasm.^90^ These approaches have a positive effect on adoption levels and underline the need for open policies and public consultation.

## 7 Future Scope and Recommendation

The potential future of e-fuels in clean transport is premised on ongoing technology development, policy frameworks, and economic scale-up. Transportation sectors in need of decarbonization, however, are now seeking e-fuels as an alternative to conventional fossil fuels, especially for hard-to-electrify markets like shipping, aviation, and heavy transport. Large-scale use is hampered by high production costs, energy efficiency, and infrastructure issues. The focus of future studies should be on improving the efficiency of electrolysis, carbon capture processes, and use of renewable sources to make e-fuels competitive and affordable.^110^ Hybrid energy systems involving e-fuels and battery-electric and hydrogen-based technologies can establish a more dynamic and robust transport system. Policy and regulation support will play a crucial role in shaping the e-fuels of the future. Governments and international institutions must establish clear incentives, carbon pricing mechanisms, and subsidies to trigger investment in the production of e-fuels. Streamlining regulations and certification processes for e-fuels can make market adoption rapid. Infrastructure development like refueling stations, storage terminals, and distribution networks will also be required to facilitate smooth incorporation into established supply chains.^45,85^ Scaling up pilot programs, encouraging public-private partnerships, and creating life cycle emission studies in order to ascertain the long-term environmental benefit of e-fuels should be prioritized by stakeholders to promote large-scale uptake. Investment in research and innovation, combined with policy interventions, will be necessary to make e-fuels a major driver in global decarbonization of transportation.^111^ The support and the necessary advancements will provide e-fuels with the proper role in bridging net-zero-emission objectives without compromising either energy security or operational efficiency.

## 8 Conclusion

Decarbonizing transport is crucial to meeting global climate objectives. This review uniquely targets electrofuels as a solution for hard-to-electrify sectors such as aviation, maritime, rail, and heavy-duty road transport. The review study addresses strategic relevance and practical challenges at the techno-economic, technology pathway, and system-level integration with existing combustion infrastructure.

The review further identifies the current production cost of e-fuel at 3–6 EUR/L for e-diesel and 2–5 EUR/L for e-methanol, driven largely by the high energy intensity of the processes involved, which require up to 50 kWh of renewable electricity per liter of fuel. Efficiency metrics have vastly improved due to recent technological advancements: optimized pathways can achieve energy conversion efficiencies up to 70% or even nearing 80% with catalytic CO_2_ hydrogenation pathways for e-kerosene options. These pathways are expected to deliver reductions of 75–90% in life cycle GHG emissions when powered by low-carbon electricity, compared with fossil fuels. Large-scale renewable energy use, process improvements, and supportive policy instruments are likely to reduce production costs to approximately 1.53 EUR per liter by 2030, particularly for applications in aviation and maritime transport. In aggregate, these quantitative results show that, though they require substantial renewable electricity and CO_2_ supply infrastructure, e-fuels provide a valid transitional solution for hard-to-abate transport sectors, at least under realistic techno-economic pathways.

In the future, progress in deploying renewables in the power sector, improving the efficiency of electrolysis technology, and advancing CO_2_ capture, combined with supportive carbon pricing, can help trigger the large-scale production of e-fuels for hard-to-abate transportation segments. In that way, e-fuels can move from being a technology transition to becoming a significant long-term part of a resilient, low-carbon transportation system.

## Data Availability

The data underlying this study are available from the corresponding author upon reasonable request.
